# Prevalence and genotyping identification of *Cryptosporidium* in adult ruminants in central Iran

**DOI:** 10.1186/s13071-019-3759-2

**Published:** 2019-10-30

**Authors:** Zohre Firoozi, Alireza Sazmand, Alireza Zahedi, Akram Astani, Ali Fattahi-Bafghi, Narges Kiani-Salmi, Behnam Ebrahimi, Arefeh Dehghani-Tafti, Una Ryan, Fateme Akrami-Mohajeri

**Affiliations:** 1 Zoonotic Diseases Research Center, School of Public Health, Shahid Sadoughi University of Medical Sciences and Health Services, Yazd, Iran; 2 Department of Food Hygiene and Safety, School of Public Health, Shahid Sadoughi University of Medical Sciences and Health Services, Yazd, Iran; 3 Department of Pathobiology, Faculty of Veterinary Sciences, Bu-Ali Sina University, Hamedan, Iran; 4 Vector and Water-Borne Pathogens Research Group, College of Science, Health, Engineering and Education, Murdoch University, Murdoch, WA 6150 Australia; 5 Department of Microbiology Sciences, School of Medicine, Shahid Sadoughi University of Medical Sciences and Health Services, Yazd, Iran; 6 Department of Parasitology, School of Medicine, Shahid Sadoughi University of Medical Sciences and Health Services, Yazd, Iran; 7 Yazd Cardiovascular Research Center, Shahid Sadoughi University of Medical Sciences and Health Services, Yazd, Iran; 8 Department of Biostatics and Epidemiology, School of Public Health, Shahid Sadoughi University of Medical Sciences and Health Services, Yazd, Iran

**Keywords:** Cryptosporidiosis, Ruminants, Zoonosis, *18S*, *gp60*

## Abstract

**Background:**

Apicomplexan parasites of the genus *Cryptosporidium* infect a wide range of animal species as well as humans. *Cryptosporidium* spp. can cause life threatening diarrhea especially in young animals, children, immunocompromised patients and malnourished individuals. Asymptomatic cryptosporidial infections in animals can also occur, making these animals potential reservoirs of infection.

**Methods:**

In the present study, a molecular survey of *Cryptosporidium* spp. in ruminants that were slaughtered for human consumption in Yazd Province, located in central Iran was conducted. Faeces were collected per-rectum from 484 animals including 192 cattle, 192 sheep and 100 goats. DNA was extracted from all samples and screened for *Cryptosporidium* by PCR amplification of the *18S* rRNA gene. Positives were Sanger sequenced and further subtyped by sequence analysis of the 60 kDa glycoprotein (*gp60*) locus.

**Results:**

In total, *Cryptosporidium* spp. were detected in 22 animals: *C. andersoni* and *C. bovis* in seven and two cattle faecal samples, respectively, *C. ubiquitum* in five sheep, and *C. xiaoi* in six sheep and two goat samples, respectively. To our knowledge, this study provides for the first time, molecular information concerning *Cryptosporidium* species infecting goats in Iran, and is also the first report of *C. ubiquitum* and *C. xiaoi* from ruminants in Iran.

**Conclusion:**

The presence of potentially zoonotic species of *Cryptosporidium* in ruminants in this region may suggest that livestock could potentially contribute to human cryptosporidiosis, in particular among farmers and slaughterhouse workers, in the area. Further molecular studies on local human populations are required to more accurately understand the epidemiology and transmission dynamics of *Cryptosporidium* spp. in this region.

## Background

Parasites of the genus *Cryptosporidium* are ubiquitous zoonotic pathogens of humans and animals and are responsible for significant number of water-borne outbreaks worldwide [1, 2]. *Cryptosporidium* spp. infect a wide range of mammals and also birds, amphibians, fishes and reptiles [3]. Of the 39 valid species, over 20 *Cryptosporidium* species and genotypes have been identified in human patients causing asymptomatic or mild to severe gastrointestinal disease. Of these, *C. parvum* and *C. hominis* are by far the most common etiological agents responsible for cryptosporidiosis in humans worldwide [4–6]. It has been shown that several *Cryptosporidium* species are not host-specific and can infect a wide host range [7].

In Iran, various studies have reported *Cryptosporidium* in humans [8], cattle and calves [9], sheep and goats [10], water buffaloes [11], camels [12], dogs [13], cats [14], horses [15], birds [16, 17], rodents [18], vegetables [19], wastewater [20] and recreational water [21]. There are also reports of simultaneous detection of *Cryptosporidium* in livestock and people associated with them such as farmers, shepherds and slaughterhouse workers, suggesting zoonotic transmission of *Cryptosporidium* spp. from animals to humans [22–25]. However, most of the studies focused on humans and animals with diarrhea. Although livestock can play a major role as a source of human cryptosporidiosis, not all of the infected livestock show clinical signs such as diarrhea [26]. Moreover, cross-contamination of raw meat with animal excreta in the process of slaughtering is a risk factor for human cryptosporidiosis [27, 28]. To date, there is no information about infection of ruminants with *Cryptosporidium* in Yazd Province, Iran. Therefore, the aim of this study was to use molecular tools to characterize *Cryptosporidium* spp. in livestock (sheep, goats and cattle) at a local abattoir.

## Methods

### Study area

Yazd Province (32.1006°N, 54.4342°E) is located in the center of Iran, in the heart of the Dasht-e Kavir desert. Yearly rainfall of less than 100 mm and frequent summer temperatures of above 40 °C has made it one of the driest major regions in Iran. With about 152,000 cattle, 406,000 sheep, 406,000 goats and 16,000 camels, this province has one of the lowest population of livestock in the country [29]. Intensive farming is common although there are few grazing areas for small ruminants in this region. Two major sources of water for raising livestock in the region are underground aquifers and urban water supply network. Prior to the present study, no information was available concerning *Cryptosporidium* species in cattle, sheep and goats in this province.

### Collection of samples

From June to November 2017, faecal samples were collected per-rectum from 484 slaughtered animals at the Moein Dam industrialized abattoir. The sample size for the present study (192 cattle, 192 sheep and 100 goats) was calculated using EpiTools [30], based on the most recent enumeration of livestock in the region and published articles on the prevalence of *Cryptosporidium* in neighboring areas. Metadata such as age, sex, health status of animals and faecal consistency for each sample was recorded systematically. Samples were collected into individual plastic containers, placed in polystyrene foam containers beside dry ice storage boxes and shipped to Shahid Sadoughi University of Medical Sciences and Health Services, Yazd, Iran within few hours.

### DNA extraction

Faeces were thoroughly homogenized using disposable wooden applicators, three freeze/thaw cycles were preformed, and the homogenates were vortexed with sterile glass beads. Genomic DNA (gDNA) was extracted using a DNA Blood and Tissues® extraction kit (MBST, Tehran, Iran). The DNA yield was assessed using a Thermo Scientific™ NanoDrop™ spectrophotometer (Thermo Fisher Scientific, Massachusetts, USA).

### PCR amplification

All samples were screened for the presence of *Cryptosporidium* spp. at the *18S* rRNA locus using a nested-PCR producing a ~ 611-bp product [31], with modified PCR conditions as previously described [32]. All samples positive for *Cryptosporidium* spp. at the *18S* locus were further subtyped at the 60 kDa glycoprotein (*gp60*) locus using a nested-PCR producing a ~ 948-bp secondary product, as previously described [33].

### Sequencing and molecular analysis

The amplified DNA from secondary PCR products was separated by gel electrophoresis and sent for sequencing using an ABI 3730XL DNA Analyzer at the Bioneer Company (Daejeon, Republic of Korea). Sanger sequencing chromatogram files were imported into Geneious Pro 8.1.6 [34], edited, analyzed, and aligned with reference sequences from GenBank using Clustal W (http://www.clustalw.genome.jp).

### Statistical analyses

The prevalence of *Cryptosporidium* in faecal samples collected from each host species was expressed as the percentage of samples positive at the *18S* locus, with 95% confidence intervals calculated assuming a binomial distribution, using the software Quantitative Parasitology 3.0 [35]. Chi-square and Fisher’s exact tests were performed using SPSS 20 for Windows (SPSS Inc. Chicago, USA), to determine if there was a statistical difference in the prevalence of *Cryptosporidium* between livestock species, their age and sex. *P*-values < 0.05 were considered statistically significant.

## Results

### Health status of animals and faeces consistency

Faecal samples were classified as non-formed faeces (pasty/watery; diarrheic) or formed faeces (normal; non-diarrheic). All of the faecal samples collected were formed (96.6%; 95% CI: 94.9–98.2%) with the exception of 15 (five diarrheal, four semi-liquid and six pasty). Of 194 cattle samples examined, one had mastitis and one was pregnant. All the sheep and goats appeared to be healthy.

### Prevalence of *Cryptosporidium*

Based on identification of *Cryptosporidium* at the *18S* rRNA locus, the overall prevalence in the examined animals was 4.5% (22/484; 95% CI: 2.62–6.38%) comprising a prevalence of 4.7% (9/192; 95% CI: 1.71–7.69%) in cattle, 5.7% (11/192; 95% CI: 2.42–8.97%) in sheep and 2% (2/100; 95% CI: 0–4.74%) in goats.

Of the 22 faecal samples positive for *Cryptosporidium*, the consistency of all the samples were formed. Among 261 male animals, 15 were infected with *Cryptosporidium* (5.7%; 95% CI: 2.89–8.50%), and from 221 female animals, seven were positive for *Cryptosporidium* (3.1%; 95% CI: 0.82–5.38%) however, no significant correlation between cryptosporidiosis and sex of the animals was found. Cryptosporidiosis was more prevalent in younger animals (*P* = 0.019). For the prevalence of *Cryptosporidium* in faecal samples from livestock categorized by livestock species, sex and age groups, see Additional file 1: Table S1 and Additional file 2: Table S2.

### *Cryptosporidium* species and subtypes

Sequence analysis at the *18S* locus identified *C. andersoni* (*n* = 7) and *C. bovis* (*n* = 2) in cattle, *C. ubiquitum* (*n* = 5) and *C. xiaoi* (*n* = 6) in sheep and *C. xiaoi* (*n* = 2) in goats. Out of five samples positive for *C. ubiquitum* at the *18S* locus, two were successfully subtyped at the *gp60* locus (subtype family XIIa).

The sequences obtained from 22 livestock faeces in the present study were deposited in the GenBank database under the accession numbers MN153790–MN153794, MN394767–MN394783, MK797741 and MK801770 (Additional file 3: Table S3).

## Discussion

In the present study, *Cryptosporidium* parasites were detected in the faeces of livestock that were slaughtered for human consumption in Yazd Province, central Iran, using molecular techniques. The prevalence was 4.5%, 5.7% and 2% in cattle, sheep and goats, respectively.

Since the first report of cryptosporidiosis in Iran in 1984 [36] numerous studies have reported different *Cryptosporidium* species in a wide range of mammalian hosts including *C. parvum*, *C. muris*, *C. meleagridis*, *C. wrairi* and *C. hominis* from humans [37–40], *C. parvum* from rats [41], *C. baileyi* from poultry and turkeys [9, 16], *C. parvum* from dogs [13], *C. parvum*, *C. hominis*, *C. canis* and *Cryptosporidium* pig genotype from recreational water [21, 42] and *C. andersoni* and *C. xiaoi* from livestock wastewater samples [20]. In Iran, it is estimated that 3.6% of children, 2.9% of healthy people, 1.3% of gastroenteritis patients and 4.5% of immunocompromised individuals are infected with *Cryptosporidium* species [8].

The present study provides the first insight into *Cryptosporidium* species infecting goats in Iran and is the first report of *C. xiaoi* in goats (prevalence of 2%, 95% CI: 0–4.74%). Worldwide, *C. xiaoi* and *C. ubiquitum* are responsible for over 90% of cryptosporidial infections in sheep and goats [7]. In Iran however, there is little information about caprine cryptosporidiosis, with all previous studies based on morphology alone, reporting prevalences ranging between 2.5–17.6% [43]. Studies in other countries have reported that 7.7% of healthy adult goats in Spain were infected with *Cryptosporidium* [44] and in France, a higher prevalence of *Cryptosporidium* (20%) was detected in goats aged 1–7 years in farms that had a previous history of diarrhoeal disease compared to farms that did not (6.6%) [45]. *Cryptosporidium xiaoi* has previously been reported in lambs, sheep, goats, yaks and kangaroos [46, 47] and in the present study, *C. xiaoi* was detected in adult goats aged 2 and 3 years-old, which is in contrast to previous reports of this species in goat kids [47, 48]. Further molecular-based studies are needed in Iran and other regions for better understanding of species infecting goats.

In sheep, *C. xiaoi* (*n* = 6) and *C. ubiquitum* (*n* = 5) were identified in the present study (11/192, 5.7%). These two species are responsible for over 90% of cryptosporidial infections in sheep and goats worldwide [7]. This is the first report of *C. ubiquitum* in Iran. Previous studies in sheep in Iran based on microscopy, have reported prevalences of 6.7–17.2% [49]. In the only molecular-based study, 22 (1.5%) of 1485 sheep faecal samples from Tehran city, were positive by microscopy and were typed as *C. andersoni* (*n* = 20) and *C. parvum* (*n* = 2) [50]. *Cryptosporidium ubiquitum* is a zoonotic parasite with a very wide host range [33, 51]. Infection of humans with *C. ubiquitum* has been reported primarily in industrialized nations and *C. ubiquitum* has also been detected in water sources, stormwaters and wastewaters in many geographical locations [33].

In the present study *C. andersoni* was detected in seven cattle faecal specimens (3.6% in 192; 95% CI: 0.98–6.23%) and *C. bovis* in two (1.04% in 192; 95% CI: 0.39–2.47%). In Iran, to date, *C. parvum*, *C. bovis*, *C. andersoni*, *C. muris*, *C. wrairi*, *C. serpentis*, and *C. baileyi* have been identified in calves and cattle using molecular tools [37, 39, 52–54]. *Cryptosporidium parvum* and *C. bovis* are the most commonly reported species infecting cattle worldwide, followed by *C. ryanae* and *C. andersoni*, with a few reports of *C. occultus*, *C. ubiquitum* and *C. xiaoi* [7]. However, previous studies on cattle in Iran, suggest that *C. parvum* and *C. andersoni* are the dominant species.

In this study cryptosporidiosis was more prevalent in younger animals. Age is often a significant variable for cryptosporidiosis status and etiology, and an age-related pattern in distribution of *Cryptoposridium* species has been observed in cattle, i.e. *C. parvum* is the predominant species in pre-weaned, monogastric calves up to 2 months of age, *C. bovis* and *C. ryanae* in older calves and young stock, whereas *C. andersoni* is mainly found in young stock and adult cattle [55]. In small ruminants however, species distribution differs between studies and between age groups within studies [55, 56]. Further studies in Iranian livestock of all ages including both symptomatic and asymptomatic individuals are required to better understand the associations between age, species/genotypes of *Cryptosporidium* and clinical outcome of cryptosporidiosis.

Faecal consistency of *Cryptosporidium-*infected adult ruminants in the present study was normal however, evidence supports an association between clinical disease and certain *Cryptosporidium-*species in livestock. In cattle, *C. parvum* often results in acute enteritis and pasty to watery diarrhea that in some cases lead to mortality from dehydration; *C. andersoni* is not associated with overt clinical signs but results in reduced milk production and weight gain; and infections with *C. bovis* and *C. ryanae* have not been associated with illness [56]. In small ruminants however, more research is needed to determine if species/genotype effects are significantly associated with production outcomes. Nevertheless, based on the current knowledge, *C. parvum* is more likely to be found in clinically ill lambs, whereas *C. xiaoi* and *C. ubiquitum* are more likely to be found in healthy lambs although the latter two species have been found in mild to severe diarrhoeal cases [55, 56].

In the present study *C. ubiquitum*, *C. andersoni* and *C. bovis,* all of which have zoonotic potential were found in adult ruminants. Studies suggest that the overall prevalence of *Cryptosporidium* declines with increasing age [57]. However, a study in England and Wales identified *Cryptosporidium* in 18.6% of adult cattle and 26.1% of adult sheep [58]. In Spain, *Cryptosporidium* prevalence in healthy adult cows was 8.4%, in sheep 5.3%, and in goats 7.7%, suggesting that asymptomatic adults can act as reservoirs for spreading oocysts in herds and pose a potential health risk for humans [44]. Although the prevalence of infection in the present study was low, the potential exists for contamination of water sources and for meat at slaughter and also abattoir wastewater, which is of public health concern.

## Conclusions

To our knowledge, this study reports for the first time *C. ubiquitum* in Iran and *C. xiaoi* from ruminants in the country. The presence of zoonotic species *C. ubiquitum*, *C. andersoni* and *C. bovis* may suggest that livestock could contribute to human cryptosporidiosis, in particular among farmers and slaughterhouse workers, in the area. Further molecular studies in human populations is required to more accurately understand the epidemiology and transmission dynamics of *Cryptosporidium* spp. in this region.

## Supplementary information

**Additional file 1: Table S1.** Prevalence of *Cryptosporidium* in livestock faecal samples by PCR at the *18S* rRNA gene and in males and females.

**Additional file 2: Table S2.** Prevalence of *Cryptosporidium* in livestock faecal samples by PCR at the *18S* rRNA gene categorized by age.

**Additional file 3: Table S3.** *Cryptosporidium* species in faeces of ruminants from Yazd, Iran.

## Data Availability

All data generated or analyzed during this study are included in this published article. The newly generated sequences were submitted to the GenBank database under the accession numbers MN153790-MN153794, MN394767-MN394783, MK797741 and MK801770.

## References

[1] Baldursson S, Karanis P (2011). Waterborne transmission of protozoan parasites: review of worldwide outbreaks–an update 2004–2010. Water Res..

[2] Alsmark C, Nolskog P, Angervall AL, Toepfer M, Winiecka-Krusnell J, Bouwmeester J (2018). Two outbreaks of cryptosporidiosis associated with cattle spring pasture events. Vet Parasitol Reg Stud Rep.

[3] Zahedi A, Paparini A, Jian F, Robertson I, Ryan U (2016). Public health significance of zoonotic *Cryptosporidium* species in wildlife: critical insights into better drinking water management. Int J Parasitol Parasites Wildl..

[4] Ryan U, Paparini A, Monis P, Hijjawi N (2016). It’s official—*Cryptosporidium* is a gregarine: what are the implications for the water industry?. Water Res..

[5] Xiao L, Cama VA, Ortega YR, Sterling CR (2018). *Cryptosporidium* and cryptosporidiosis. Foodborne parasites.

[6] Ryan U, Zahedi A, Paparini A (2016). *Cryptosporidium* in humans and animals—a one health approach to prophylaxis. Parasite Immunol..

[7] Feng Y, Ryan UM, Xiao L (2018). Genetic diversity and population structure of *Cryptosporidium*. Trends Parasitol..

[8] Berahmat R, Spotin A, Ahmadpour E, Mahami-Oskouei M, Rezamand A, Aminisani N (2017). Human cryptosporidiosis in Iran: a systematic review and meta-analysis. Parasitol Res..

[9] Meamar AR, Guyot K, Certad G, Dei-Cas E, Mohraz M, Mohebali M (2007). Molecular characterization of *Cryptosporidium* isolates from humans and animals in Iran. Appl Environ Microbiol..

[10] Fasihi Harandi M, Fotouhi Ardakani R (2008). *Cryptosporidium* infection on sheep and goats in Kerman: epidemiology and risk factor analysis. J Fac Vet Med Univ Tehran.

[11] Bahrami S, Alborzi AR, Molayan PH, Purbaram S, Mousavi B (2014). prevalence of *Cryptosporidium* spp. infection and its association with diarrhea in buffalo calves in Khuzestan, a southwestern province of Iran. Buffalo Bull..

[12] Sazmand A, Joachim A (2017). Parasitic diseases of camels in Iran (1931–2017)—a literature review. Parasite..

[13] Ranjbar R, Mirhendi H, Izadi M, Behrouz B, Mohammadi Manesh R (2018). Molecular identification of *Cryptosporidium* spp. in Iranian dogs using seminested PCR: a first report. Vector Borne Zoonotic Dis..

[14] Mirzaghavami M, Sadraei J, Forouzandeh M (2016). Detection of *Cryptosporidium* spp. in free ranging animals of Tehran, Iran. J Parasit Dis..

[15] Ghadrdan AR, Hamidinejat H, Alizadehnia P (2013). A Survey on frequency of equine cryptosporidiosis in Ahvaz. J Vet Clin Pathol.

[16] Hamidinejat H, Jalali MHR, Jafari RA, Nourmohammadi K (2014). Molecular determination and genotyping of *Cryptosporidium* spp. in fecal and respiratory samples of industrial poultry in Iran. Asian Pac J Trop Dis..

[17] Radfar MH, Asl EN, Seghinsara HR, Dehaghi MM, Fathi S (2012). Biodiversity and prevalence of parasites of domestic pigeons (*Columba livia domestica*) in a selected semiarid zone of South Khorasan, Iran. Trop Anim Health Prod..

[18] Gholipoury M, Rezai HR, Namroodi S, Khazaeli FA (2016). Zoonotic and non-zoonotic parasites of wild rodents in Turkman Sahra, northeastern Iran. Iran J Parasitol..

[19] Fallah AA, Makhtumi Y, Pirali-Kheirabadi K (2016). Seasonal study of parasitic contamination in fresh salad vegetables marketed in Shahrekord, Iran. Food Control..

[20] Hatam-Nahavandi K, Mohebali M, Mahvi A-H, Keshavarz H, Najafian H-R, Mirjalali H (2016). Microscopic and molecular detection of *Cryptosporidium andersoni* and *Cryptosporidium xiaoi* in wastewater samples of Tehran Province, Iran. Iran J Parasitol..

[21] Naeini KM, Asadi M, Chaleshtori MH (2011). Detection and molecular characterization of *Cryptosporidium* species in recreational waters of Chaharmahal va Bakhtiyari Province of Iran using nested-PCR-RFLP. Iran J Parasitol..

[22] Sazmand A, Rasooli A, Nouri M, Hamidinejat H, Hekmatimoghaddam S (2012). Prevalence of *Cryptosporidium* spp. in camels and involved people in Yazd Province, Iran. Iran J Parasitol..

[23] Nouri M, Toroghi R (1991). Asymptomatic cryptosporidiosis in cattle and humans in Iran. Vet Rec..

[24] Nouri M, Karami M (1991). Asymptomatic cryptosporidiosis in nomadic shepherds and their sheep. J Infect..

[25] Jafari R, Maghsood AH, Fallah M (2012). Prevalence of *Cryptosporidium* infection among livestock and humans in contact with livestock in Hamadan district, Iran, 2012. J Res Health Sci..

[26] Xiao L, Feng Y (2008). Zoonotic cryptosporidiosis. FEMS Immunol Med Microbiol..

[27] Budu-Amoako E, Greenwood SJ, Dixon BR, Barkema HW, McClure J (2011). Foodborne illness associated with *Cryptosporidium* and *Giardia* from livestock. J Food Protect..

[28] Laberge I, Griffiths MW, Griffiths M (1996). Prevalence, detection and control of *Cryptosporidium parvum* in food. Int J Food Microbiol..

[29] Ministry of Agriculture-Jahad of Iran. Annual production report. Tehran; 2018 **(in Persian)**.

[30] Sergeant E. Epitools epidemiological calculators. 2018. http://epitools.ausvet.com.au/. Accessed 4 Sept 2019.

[31] Silva SO, Richtzenhain LJ, Barros IN, Gomes AM, Silva AV, Kozerski ND (2013). A new set of primers directed to *18S* rRNA gene for molecular identification of *Cryptosporidium* spp. and their performance in the detection and differentiation of oocysts shed by synanthropic rodents. Exp Parasitol..

[32] Zahedi A, Phasey J, Boland T, Ryan U (2016). First report of *Cryptosporidium* species in farmed and wild buffalo from the Northern Territory, Australia. Parasitol Res..

[33] Li N, Xiao L, Alderisio K, Elwin K, Cebelinski E, Chalmers R (2014). Subtyping *Cryptosporidium ubiquitum*, a zoonotic pathogen emerging in humans. Emerg Infect Dis..

[34] Kearse M, Moir R, Wilson A, Stones-Havas S, Cheung M, Sturrock S (2012). Geneious Basic: an integrated and extendable desktop software platform for the organization and analysis of sequence data. Bioinformatics..

[35] Rózsa L, Reiczigel J, Majoros G (2000). Quantifying parasites in samples of hosts. J Parasitol..

[36] Gharagozlou M (1984). Report of one case of cryptosporidiosis in a calf and analysis of this disease. J Fac Vet Med Univ Tehran.

[37] Azami M, Moghaddam DD, Salehi R, Salehi M (2007). The identification of *Cryptosporidium* species in Isfahan, Iran by PCR-RFLP analysis of the *18S* rRNA gene. Mol Biol..

[38] Pirestani M, Sadraei J, Zavvar M, Vaeznia H (2008). Molecular characterization of *Cryptosporidium* isolates from human and bovine using *18S* rRNA gene in Shahriar county of Tehran, Iran. Parasitol Res..

[39] Nazemalhosseini-Mojarad E, Haghighi A, Taghipour N, Keshavarz A, Mohebi SR, Zali MR (2011). Subtype analysis of *Cryptosporidium parvum* and *Cryptosporidium hominis* isolates from humans and cattle in Iran. Vet Parasitol..

[40] Rafiei A, Rashno Z, Samarbafzadeh A, Khademvatan S (2014). Molecular characterization of *Cryptosporidium* spp. isolated from immunocompromised patients and children. Jundishapur J Microbiol..

[41] Bahrami F, Sadraei J, Frozandeh M (2012). Molecular characterization of *Cryptosporidium* spp. in wild rats of Tehran, Iran using *18S* rRNA gene and PCR-RFLP method. Jundishapur J Microbiol..

[42] Mohammadi Ghaleh Bin B, Fallah E, Asgharzadeh M, Kazemi A (2007). Detection of *Cryptosporidium* species in water resources of Ardabil Province by PCR, RFLP. J Ardabil Univ Med Sci..

[43] Mohammadzadeh A. Study on the frequency of cryptosporidiosis in lambs of Ahwaz district of Khuzestan Province. DVM Thesis, Shahid Chamran University of Ahvaz; 1993 **(in Persian)**.

[44] Castro-Hermida JA, Almeida A, González-Warleta M, da Costa JMC, Rumbo-Lorenzo C, Mezo M (2007). Occurrence of *Cryptosporidium parvum* and *Giardia duodenalis* in healthy adult domestic ruminants. Parasitol Res..

[45] Castro-Hermida J, Delafosse A, Pors I, Ares-Mazás E, Chartier C (2005). *Giardia duodenalis* and *Cryptosporidium parvum* infections in adult goats and their implications for neonatal kids. Vet Rec..

[46] Yang R, Fenwick S, Potter A, Ng J, Ryan U (2011). Identification of novel *Cryptosporidium* genotypes in kangaroos from Western Australia. Vet Parasitol..

[47] Fayer R, Santín M (2009). *Cryptosporidium xiaoi* n. sp. (Apicomplexa: Cryptosporidiidae) in sheep (*Ovis aries*). Vet Parasitol..

[48] Díaz P, Quílez J, Robinson G, Chalmers RM, Díez-Baños P, Morrondo P (2010). Identification of *Cryptosporidium xiaoi* in diarrhoeic goat kids (*Capra hircus*) in Spain. Vet Parasitol..

[49] Ahourai P, Ezzi A, Gholami M, Vandyoosefi J, Kargar R, Maalhagh N (1985). *Cryptosporidium* spp. in new born lambs in Iran. Trop Anim Health Prod..

[50] Dalimi A, Tahvildar F, Ghaffarifar F (2017). Report of *Cryptosporidium andersoni* based on HSP70 gene amplification isolated from sheep in Tehran. J Vet Microbiol.

[51] Fayer R, Santín M, Macarisin D (2010). *Cryptosporidium ubiquitum* n. sp. in animals and humans. Vet Parasitol..

[52] Fotouhi Ardakani R, Fasihi Harandi M, Solayman Banai S, Kamyabi H, Atapour M, Sharifi I (2008). Epidemiology of *Cryptosporidium* infection of cattle in Kerman/Iran and molecular genotyping of some isolates. J Kerman Univ Med Sci.

[53] Keshavarz A, Haghighi A, Athari A, Kazemi B, Abadi A, Mojarad EN (2009). Prevalence and molecular characterization of bovine *Cryptosporidium* in Qazvin province, Iran. Vet Parasitol..

[54] Mirzai Y, Yakhchali M, Mardani K (2014). *Cryptosporidium parvum* and *Cryptosporidium andersoni* infection in naturally infected cattle of northwest Iran. Vet Res Forum..

[55] Robertson LJ, Björkman C, Axén C, Fayer R, Caccio SM, Giovanni W (2014). Cryptosporidiosis in farmed animals. *Cryptosporidium*: parasite and disease.

[56] Santín M (2013). Clinical and subclinical infections with *Cryptosporidium* in animals. N Z Vet J..

[57] Fayer R, Santín M, Trout JM (2007). Prevalence of *Cryptosporidium* species and genotypes in mature dairy cattle on farms in eastern United States compared with younger cattle from the same locations. Vet Parasitol..

[58] Smith R, Chalmers R, Mueller-Doblies D, Clifton-Hadley F, Elwin K, Watkins J (2010). Investigation of farms linked to human patients with cryptosporidiosis in England and Wales. Prev Vet Med..

