# Supplementary material for: Prevalence and genotyping identification of Cryptosporidium in adult ruminants in central Iran
Source: Parasit Vectors. 2019 Oct 30;12:510. doi: 10.1186/s13071-019-3759-2 (PMC6822396; doi:10.1186/s13071-019-3759-2)
Supplement: Supplementary file 1 — Additional file 1: Table S1. Prevalence of Cryptosporidium in livestock faecal samples by PCR at the 18S rRNA gene and in males and females. [file 13071_2019_3759_MOESM1_ESM.docx]

**Additional file 1: Table S1.** Prevalence of *Cryptosporidium* in livestock faecal samples by PCR at the *18S* rRNA gene and in males and females.

| **Category** | **Number of samples examined (%)** | **Number positive (%)** | **95% CI** | **p-Value** |
| --- | --- | --- | --- | --- |
| **Livestock** | N=484 |  |  |  |
| Cattle | 192 (39.67) | 9 (4.7) | 1.71–7.69 |  |
| Sheep | 192 (39.67) | 11 (5.7) | 2.42–8.97 | 0.3 |
| Goat | 100 (20.66) | 2 (2) | 0–4.74 |  |
|  |  |  |  |  |
| **Sex** |  |  |  |  |
| Male | 247 (51.03) | 15 (6.1) | 3.12–9.08 |  |
| Female | 237 (48.97) | 7 (3) | 0.84–5.2 | 0.1 |
