# Supplementary material for: Prevalence and genotyping identification of Cryptosporidium in adult ruminants in central Iran
Source: Parasit Vectors. 2019 Oct 30;12:510. doi: 10.1186/s13071-019-3759-2 (PMC6822396; doi:10.1186/s13071-019-3759-2)
Supplement: Supplementary file 2 — Additional file 2: Table S2. Prevalence of Cryptosporidium in livestock faecal samples by PCR at the 18S rRNA gene categorized by age. [file 13071_2019_3759_MOESM2_ESM.docx]

**Additional file 2:** Table S2. Prevalence of *Cryptosporidium* in livestock faecal samples by PCR at the *18S* rRNA gene categorized by age.

| **Animals and age groups (year)** | **Number of samples examined** | **Number positive (%)** | **95% CI** | **p-Value** |
| --- | --- | --- | --- | --- |
| Cattle |  |  |  |  |
| <3 | 65 | 5 (7.7) | 1.2–14.1 |  |
| 3-4 | 46 | 3 (6.5) | 0.56–13.6 | 0.66 |
| >4 | 81 | 1 (1.2) | 1.2–3.6 |  |
| Sheep |  |  |  |  |
| <3 | 67 | 7 (10.4) | 2.8–17.7 |  |
| 3-4 | 104 | 2 (1.9) | 0.65–4.4 | 0.23 |
| >4 | 21 | 2 (9.5) | 0.48–18.5 |  |
| Goat |  |  |  |  |
| <3 | 22 | 1 (4.5) | 4.1–13.1 |  |
| 3-4 | 61 | 1 (1.6) | 1.5–4.7 | 0.07 |
| >4 | 17 | 0 | – |  |
| Total |  |  |  |  |
| <3 | 154 | 13 (8.4) | 4.1–12.7 |  |
| 3-4 | 211 | 6 (2.8) | 3.8–9.4 | 0.019 |
| >4 | 119 | 3 (2.5) | 0.24–5.2 |  |
