# Supplementary material for: Prevalence and genotyping identification of Cryptosporidium in adult ruminants in central Iran
Source: Parasit Vectors. 2019 Oct 30;12:510. doi: 10.1186/s13071-019-3759-2 (PMC6822396; doi:10.1186/s13071-019-3759-2)
Supplement: Supplementary file 3 — Additional file 3: Table S3. Cryptosporidium species in faeces of ruminants from Yazd, Iran. [file 13071_2019_3759_MOESM3_ESM.docx]

**Additional file 1:** Table S3. *Cryptosporidium* species in faeces of ruminants from Yazd, Iran

| **Sample ID** | **Host species** | **sex** | **Age (year)** | **Faecal consistency** | **Species** | **Gene locus** | **GenBank accession number** |
| --- | --- | --- | --- | --- | --- | --- | --- |
| YZD7 | cattle | female | 2.5 | Formed | *C. andersoni* | *18S* rRNA | MN153790 |
| YZD13 | cattle | female | 3 | Formed | *C. andersoni* | *18S* rRNA | MN394767 |
| YZD296 | cattle | male | 2 | Formed | *C. andersoni* | *18S* rRNA | MN394772 |
| YZD439 | cattle | female | 10 | Formed | *C. andersoni* | *18S* rRNA | MN394780 |
| YZD446 | cattle | male | 3 | Formed | *C. andersoni* | *18S* rRNA | MN394781 |
| YZD457 | cattle | male | 2 | Formed | *C. andersoni* | *18S* rRNA | MN394782 |
| YZD463 | cattle | male | 2 | Formed | *C. andersoni* | *18S* rRNA | MN394783 |
| YZD415 | cattle | male | 1 | Formed | *C. bovis* | *18S* rRNA | MN394779 |
| YZD234 | cattle | male | 3 | Formed | *C. bovis* | *18S* rRNA | MN153794 |
|  |  |  |  |  |  |  |  |
| YZD60 | sheep | male | 4 | Formed | *C. ubiquitum* | *18S* rRNA | MN153791 |
| YZD272 | sheep | male | 2 | Formed | *C. ubiquitum* | *18S* rRNA | MN394768 |
| YZD274 | sheep | male | 2 | Formed | *C. ubiquitum* | *18S* rRNA | MN394769 |
| YZD353 | sheep | female | *5* | Formed | *C. ubiquitum* | *18S* rRNA | MN394776 |
| YZD356 | sheep | male | 3 | Formed | *C. ubiquitum* | *18S* rRNA | MN394777 |
| YZD64 | sheep | male | 1 | Formed | *C. xiaoi* | *18S* rRNA | MN153792 |
| YZD284 | sheep | male | 2 | Formed | *C. xiaoi* | *18S* rRNA | MN394770 |
| YZD290 | sheep | male | 2 | Formed | *C. xiaoi* | *18S* rRNA | MN394771 |
| YZD338 | sheep | female | 2 | Formed | *C. xiaoi* | *18S* rRNA | MN394774 |
| YZD340 | sheep | female | 5 | Formed | *C. xiaoi* | *18S* rRNA | MN394775 |
| YZD362 | sheep | female | 2 | Formed | *C. xiaoi* | *18S* rRNA | MN394778 |
|  | |  |  |  |  |  |  |
| YZD317 | goat | male | 3 | Formed | *C. xiaoi* | *18S* rRNA | MN153793 |
| YZD323 | goat | male | 2 | Formed | *C. xiaoi* | *18S* rRNA | MN394773 |
|  |  |  |  |  |  |  |  |
| YZD353 | sheep | female | 5 | Formed | *C. ubiquitum* | *gp60* | MK797741 |
| YZD356 | sheep | male | 3 | Formed | *C. ubiquitum* | *gp60* | MK801770 |
